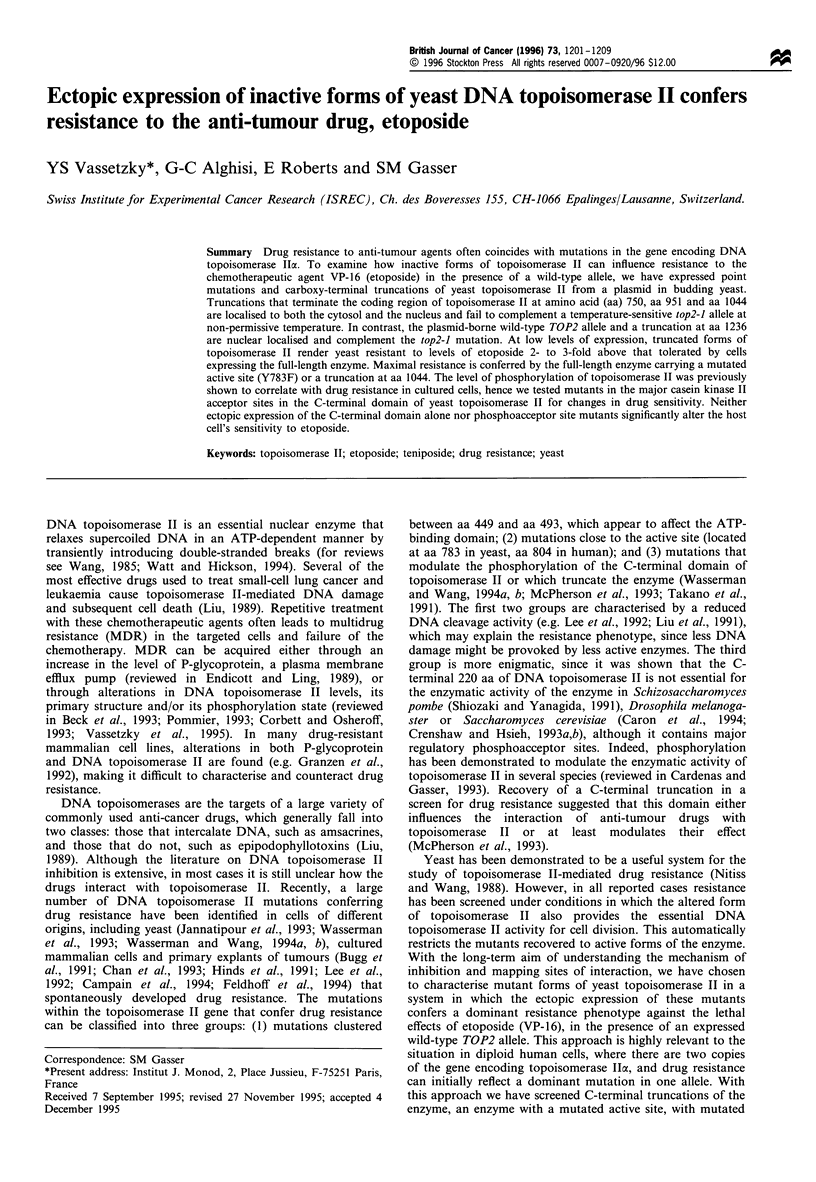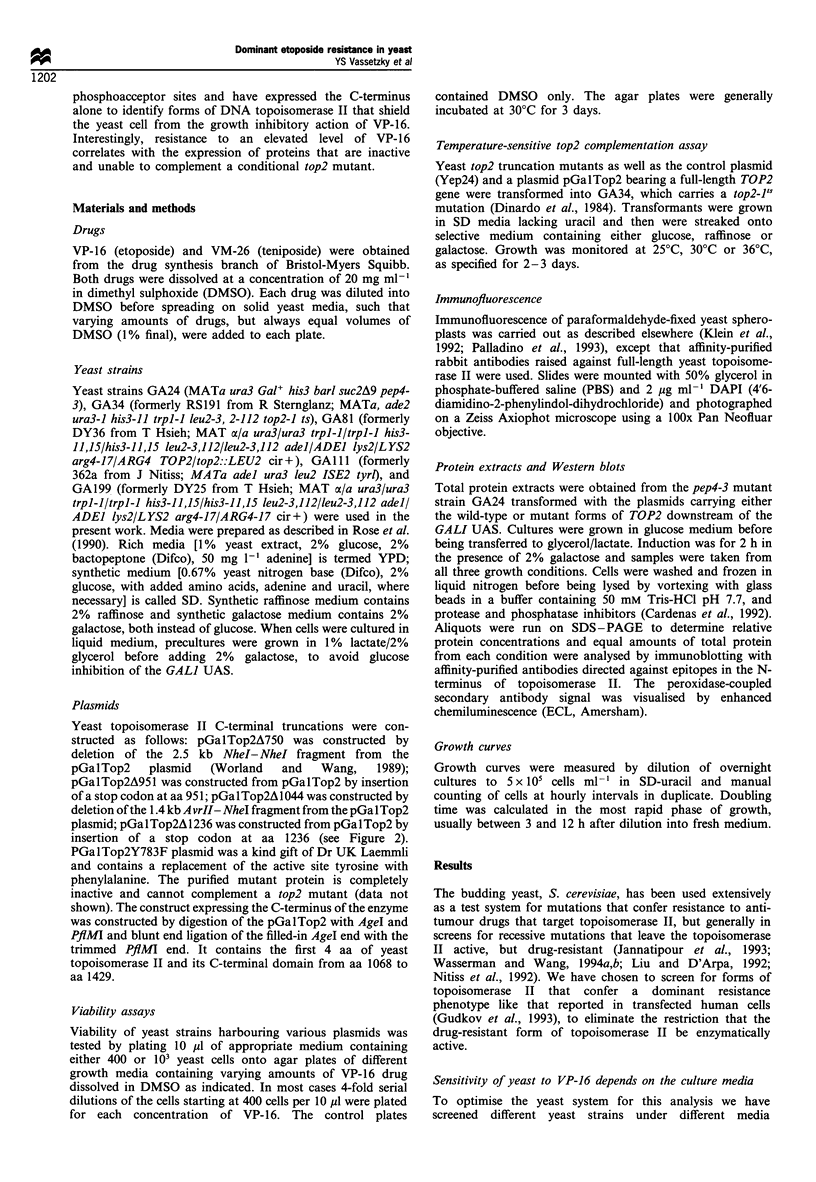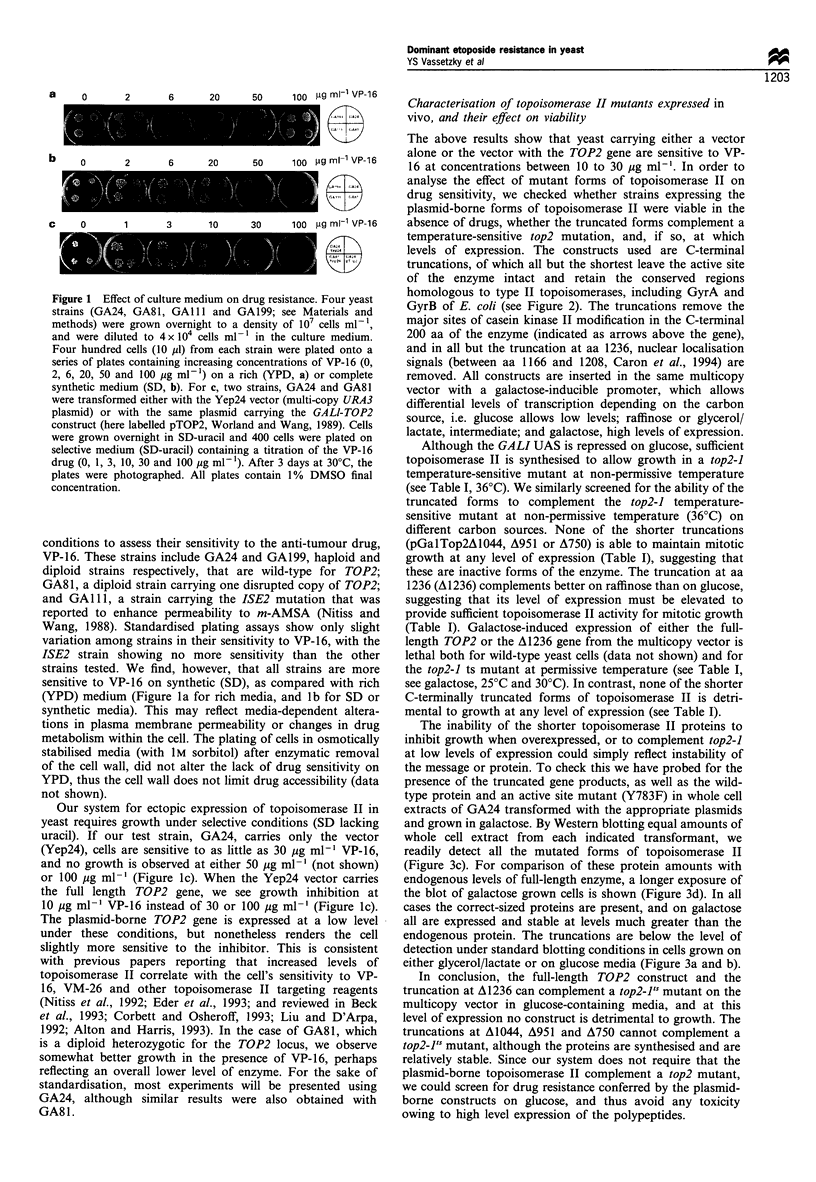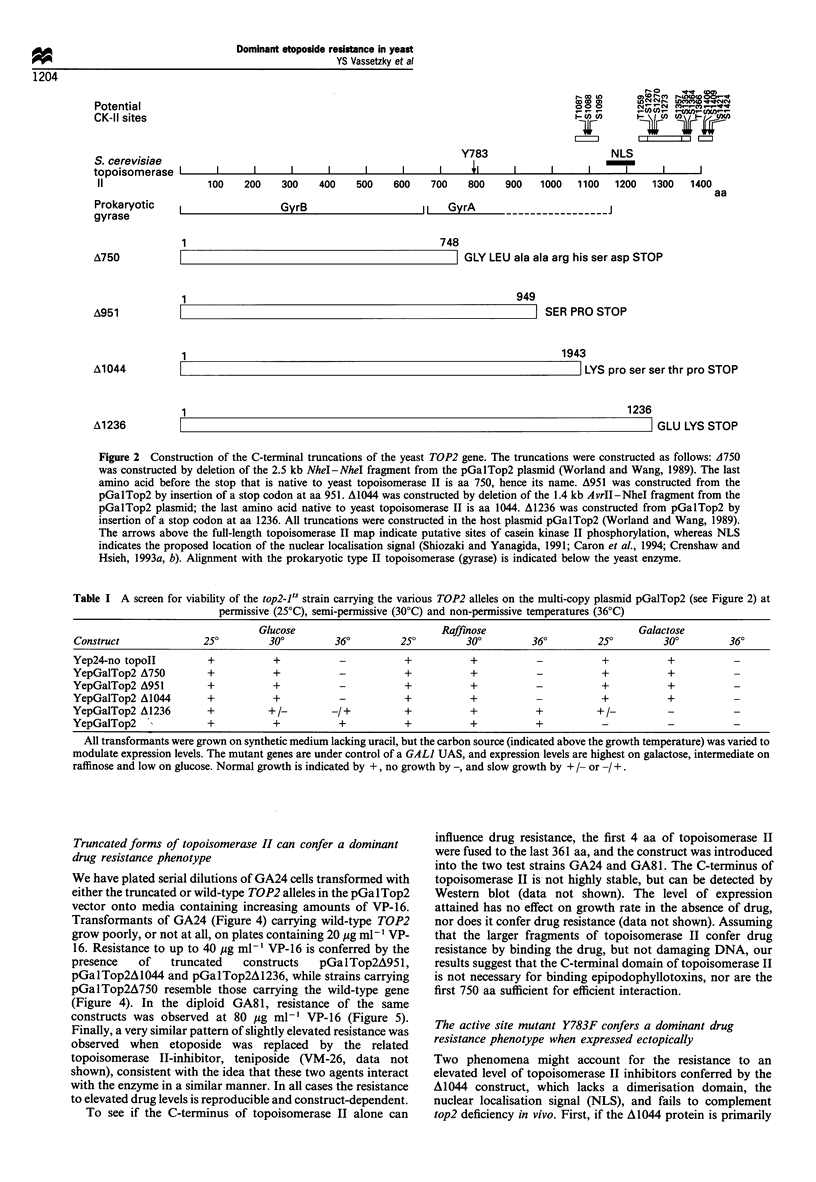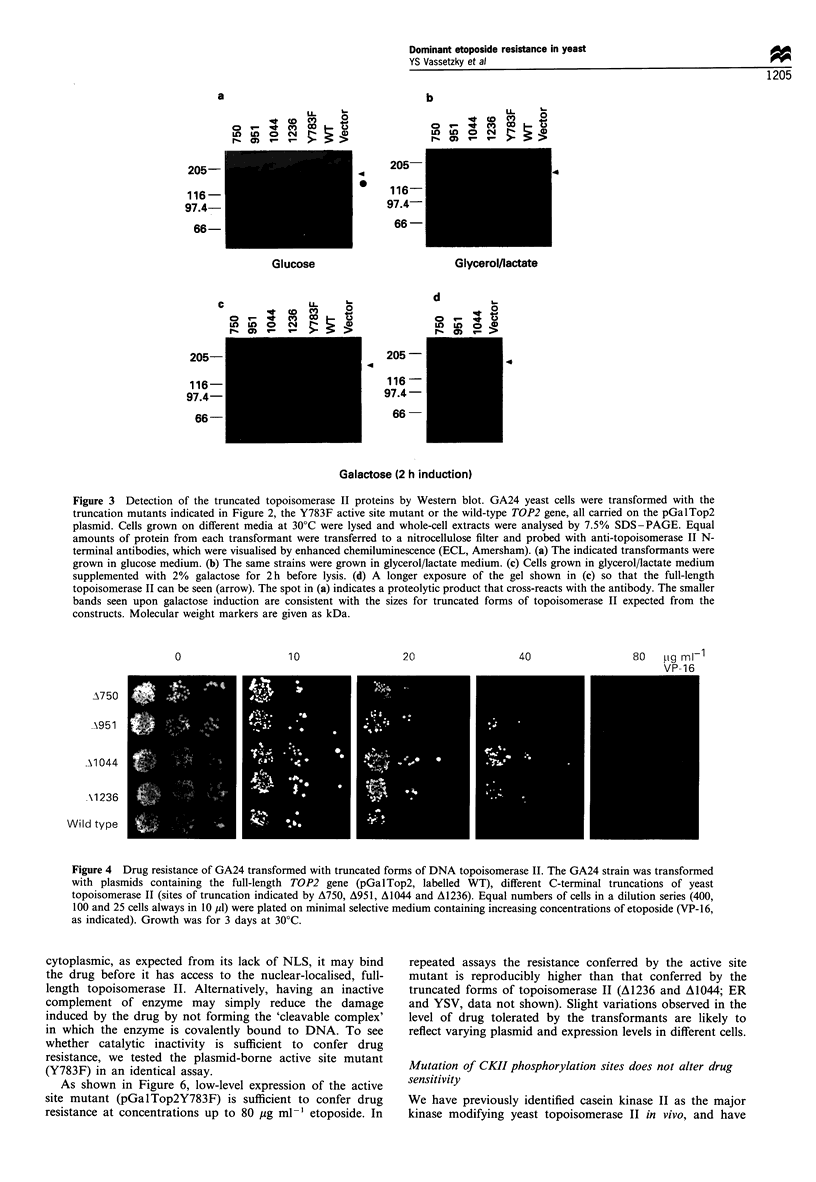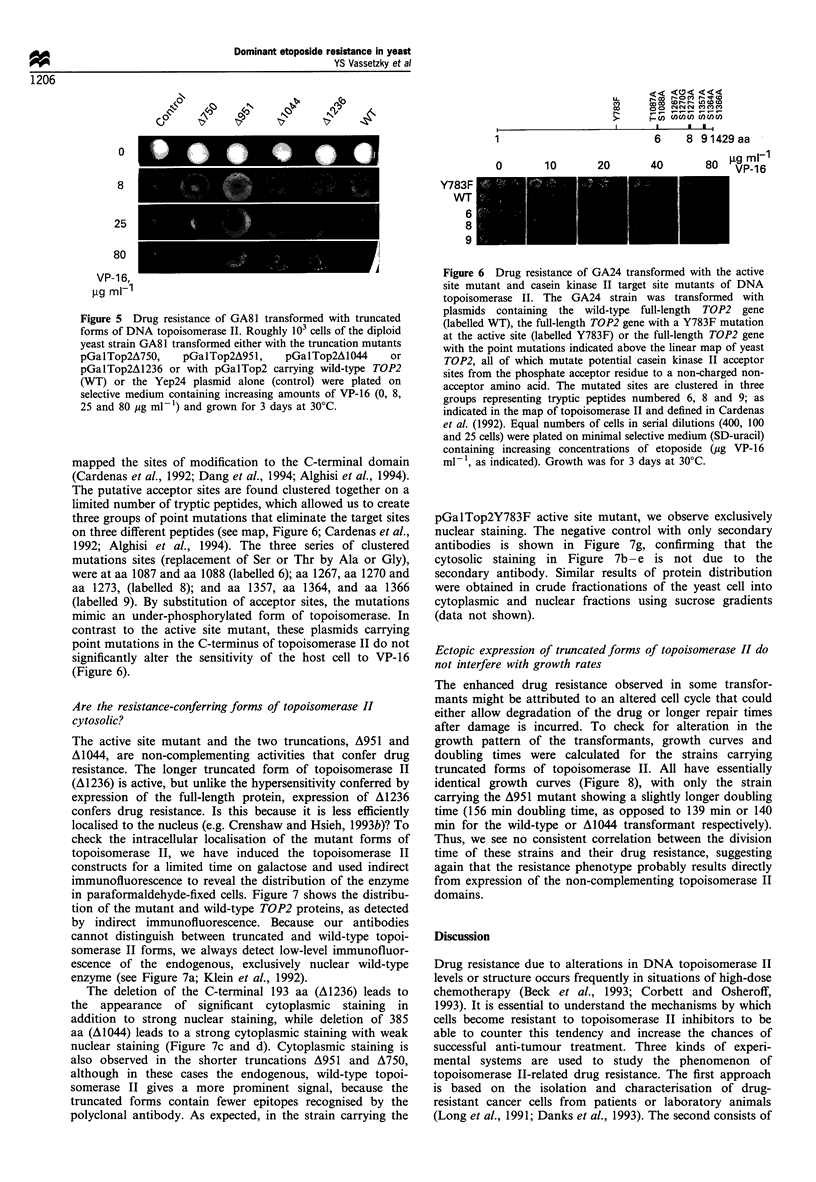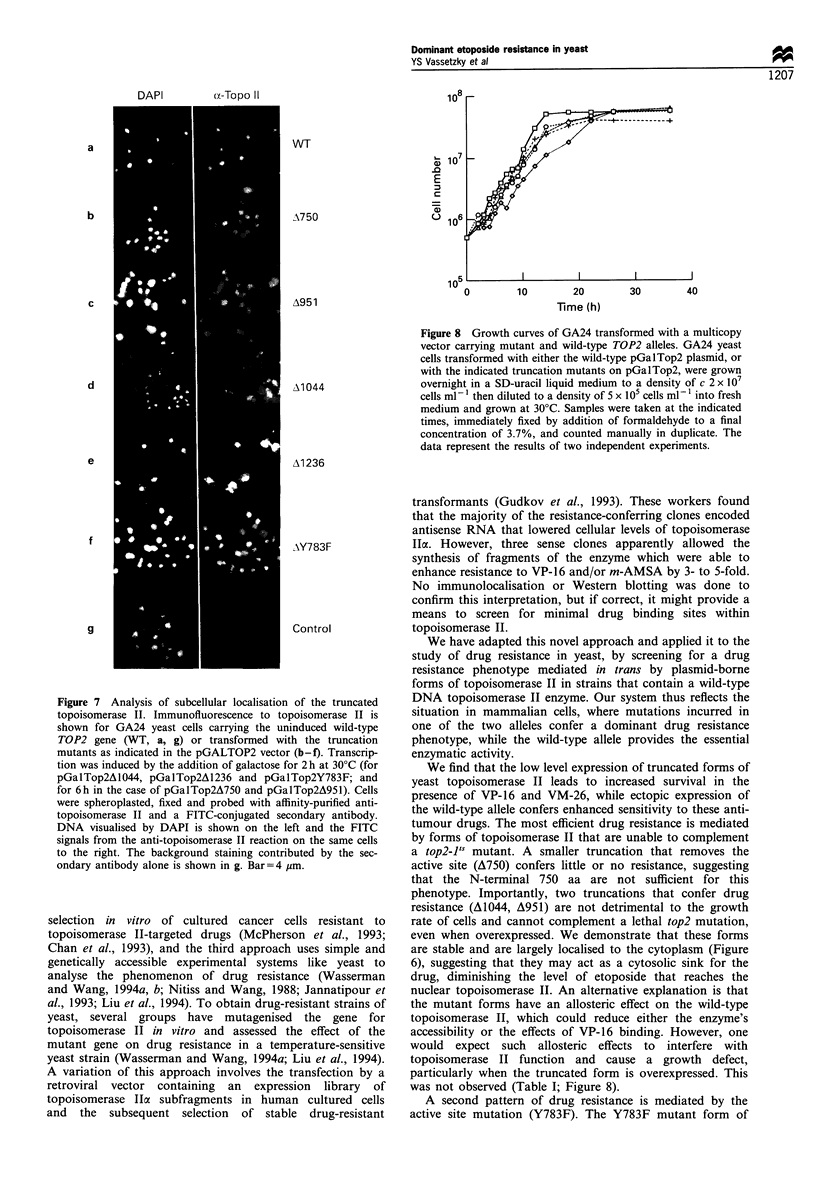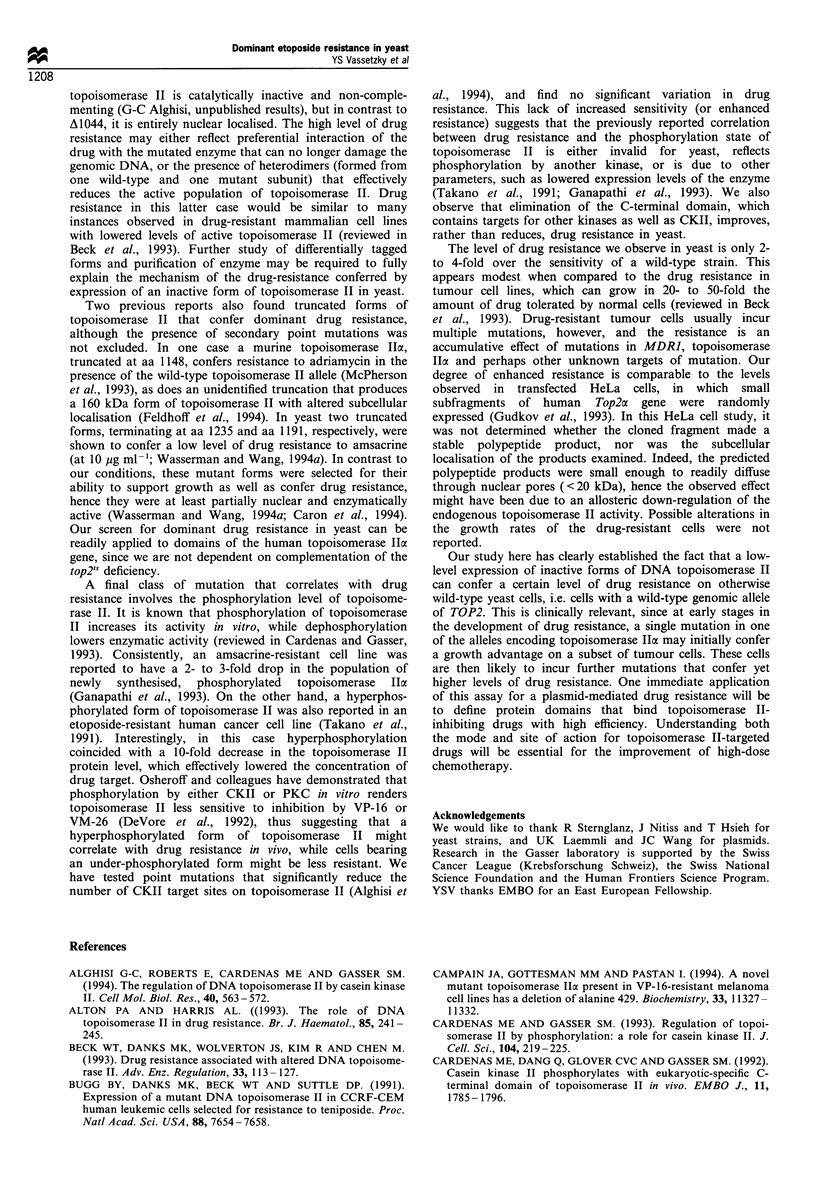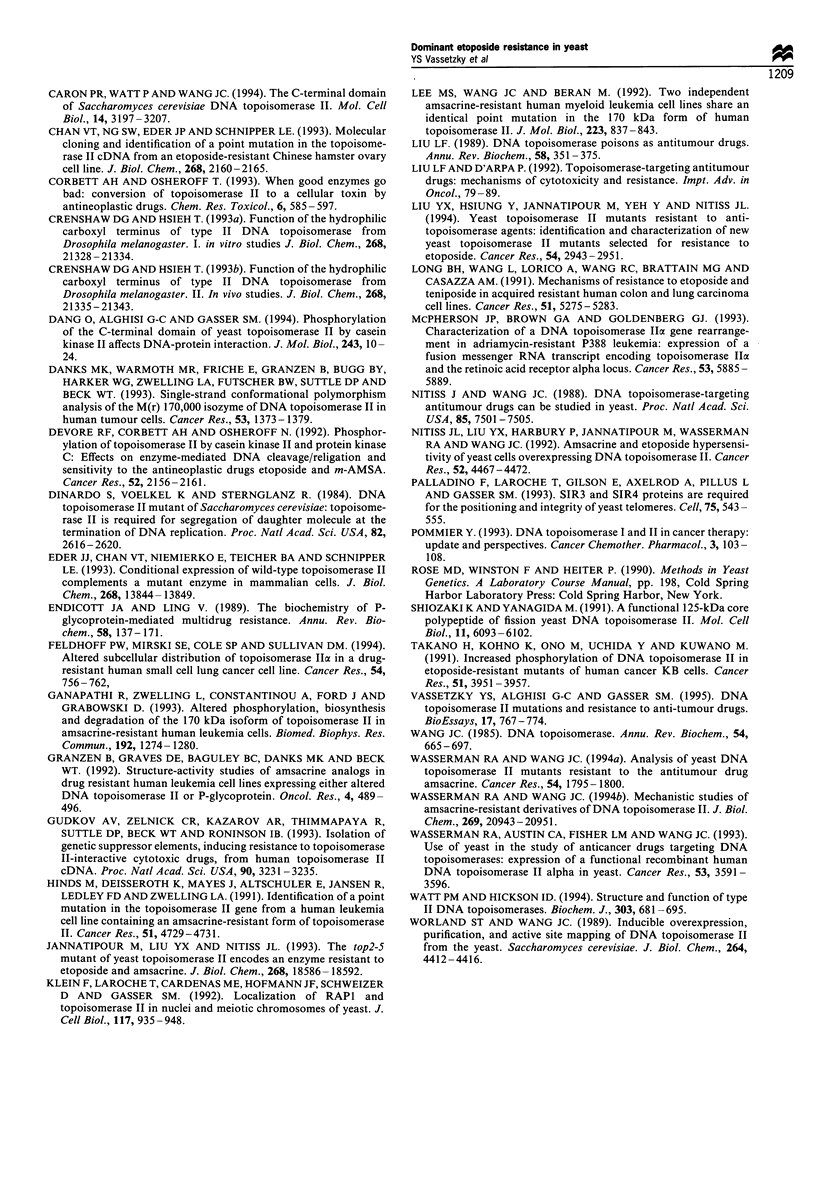# Ectopic expression of inactive forms of yeast DNA topoisomerase II confers resistance to the anti-tumour drug, etoposide.

**DOI:** 10.1038/bjc.1996.231

**Published:** 1996-05

**Authors:** Y. S. Vassetzky, G. C. Alghisi, E. Roberts, S. M. Gasser

**Affiliations:** Swiss Institute for Experimental Cancer Research (ISREC), Epalinges/Lausanne, Switzerland.

## Abstract

**Images:**


British Journal of Cancer (1996) 73, 1201–1209
© 1996 Stockton Press All rights reserved 0007–0920/96 $12.00

### Ectopic expression of inactive forms of yeast DNA topoisomerase II confers resistance to the anti-tumour drug, etoposide

YS Vassetzky*, G-C Alghisi, E Roberts and SM Gasser

*Swiss Institute for Experimental Cancer Research (ISREC), Ch. des Boveresses 155, CH-1066 Epalinges/Lausanne, Switzerland.*

**Summary** Drug resistance to anti-tumour agents often coincides with mutations in the gene encoding DNA topoisomerase IIα. To examine how inactive forms of topoisomerase II can influence resistance to the chemotherapeutic agent VP-16 (etoposide) in the presence of a wild-type allele, we have expressed point mutations and carboxy-terminal truncations of yeast topoisomerase II from a plasmid in budding yeast. Truncations that terminate the coding region of topoisomerase II at amino acid (aa) 750, aa 951 and aa 1044 are localised to both the cytosol and the nucleus and fail to complement a temperature-sensitive *top2-1* allele at non-permissive temperature. In contrast, the plasmid-borne wild-type *TOP2* allele and a truncation at aa 1236 are nuclear localised and complement the *top2-1* mutation. At low levels of expression, truncated forms of topoisomerase II render yeast resistant to levels of etoposide 2- to 3-fold above that tolerated by cells expressing the full-length enzyme. Maximal resistance is conferred by the full-length enzyme carrying a mutated active site (Y783F) or a truncation at aa 1044. The level of phosphorylation of topoisomerase II was previously shown to correlate with drug resistance in cultured cells, hence we tested mutants in the major casein kinase II acceptor sites in the C-terminal domain of yeast topoisomerase II for changes in drug sensitivity. Neither ectopic expression of the C-terminal domain alone nor phosphoacceptor site mutants significantly alter the host cell's sensitivity to etoposide.

**Keywords:** topoisomerase II; etoposide; teniposide; drug resistance; yeast

DNA topoisomerase II is an essential nuclear enzyme that relaxes supercoiled DNA in an ATP-dependent manner by transiently introducing double-stranded breaks (for reviews see Wang, 1985; Watt and Hickson, 1994). Several of the most effective drugs used to treat small-cell lung cancer and leukaemia cause topoisomerase II-mediated DNA damage and subsequent cell death (Liu, 1989). Repetitive treatment with these chemotherapeutic agents often leads to multidrug resistance (MDR) in the targeted cells and failure of the chemotherapy. MDR can be acquired either through an increase in the level of P-glycoprotein, a plasma membrane efflux pump (reviewed in Endicott and Ling, 1989), or through alterations in DNA topoisomerase II levels, its primary structure and/or its phosphorylation state (reviewed in Beck *et al.*, 1993; Pommier, 1993; Corbett and Osheroff, 1993; Vassetzky *et al.*, 1995). In many drug-resistant mammalian cell lines, alterations in both P-glycoprotein and DNA topoisomerase II are found (e.g. Granzen *et al.*, 1992), making it difficult to characterise and counteract drug resistance.

DNA topoisomerases are the targets of a large variety of commonly used anti-cancer drugs, which generally fall into two classes: those that intercalate DNA, such as amsacrines, and those that do not, such as epipodophyllotoxins (Liu, 1989). Although the literature on DNA topoisomerase II inhibition is extensive, in most cases it is still unclear how the drugs interact with topoisomerase II. Recently, a large number of DNA topoisomerase II mutations conferring drug resistance have been identified in cells of different origins, including yeast (Jannatipour *et al.*, 1993; Wasserman *et al.*, 1993; Wasserman and Wang, 1994*a*, *b*), cultured mammalian cells and primary explants of tumours (Bugg *et al.*, 1991; Chan *et al.*, 1993; Hinds *et al.*, 1991; Lee *et al.*, 1992; Campain *et al.*, 1994; Feldhoff *et al.*, 1994) that spontaneously developed drug resistance. The mutations within the topoisomerase II gene that confer drug resistance can be classified into three groups: (1) mutations clustered between aa 449 and aa 493, which appear to affect the ATP-binding domain; (2) mutations close to the active site (located at aa 783 in yeast, aa 804 in human); and (3) mutations that modulate the phosphorylation of the C-terminal domain of topoisomerase II or which truncate the enzyme (Wasserman and Wang, 1994*a*, *b*; McPherson *et al.*, 1993; Takano *et al.*, 1991). The first two groups are characterised by a reduced DNA cleavage activity (e.g. Lee *et al.*, 1992; Liu *et al.*, 1991), which may explain the resistance phenotype, since less DNA damage might be provoked by less active enzymes. The third group is more enigmatic, since it was shown that the C-terminal 220 aa of DNA topoisomerase II is not essential for the enzymatic activity of the enzyme in *Schizosaccharomyces pombe* (Shiozaki and Yanagida, 1991), *Drosophila melanogaster* or *Saccharomyces cerevisiae* (Caron *et al.*, 1994; Crenshaw and Hsieh, 1993*a,b*), although it contains major regulatory phosphoacceptor sites. Indeed, phosphorylation has been demonstrated to modulate the enzymatic activity of topoisomerase II in several species (reviewed in Cardenas and Gasser, 1993). Recovery of a C-terminal truncation in a screen for drug resistance suggested that this domain either influences the interaction of anti-tumour drugs with topoisomerase II or at least modulates their effect (McPherson *et al.*, 1993).

Yeast has been demonstrated to be a useful system for the study of topoisomerase II-mediated drug resistance (Nitiss and Wang, 1988). However, in all reported cases resistance has been screened under conditions in which the altered form of topoisomerase II also provides the essential DNA topoisomerase II activity for cell division. This automatically restricts the mutants recovered to active forms of the enzyme. With the long-term aim of understanding the mechanism of inhibition and mapping sites of interaction, we have chosen to characterise mutant forms of yeast topoisomerase II in a system in which the ectopic expression of these mutants confers a dominant resistance phenotype against the lethal effects of etoposide (VP-16), in the presence of an expressed wild-type *TOP2* allele. This approach is highly relevant to the situation in diploid human cells, where there are two copies of the gene encoding topoisomerase IIα, and drug resistance can initially reflect a dominant mutation in one allele. With this approach we have screened C-terminal truncations of the enzyme, an enzyme with a mutated active site, with mutated

Correspondence: SM Gasser
*Present address: Institut J. Monod, 2, Place Jussieu, F-75251 Paris, France
Received 7 September 1995; revised 27 November 1995; accepted 4 December 1995

phosphoacceptor sites and have expressed the C-terminus alone to identify forms of DNA topoisomerase II that shield the yeast cell from the growth inhibitory action of VP-16. Interestingly, resistance to an elevated level of VP-16 correlates with the expression of proteins that are inactive and unable to complement a conditional *top2* mutant.

### Materials and methods

### *Drugs*

VP-16 (etoposide) and VM-26 (teniposide) were obtained from the drug synthesis branch of Bristol-Myers Squibb. Both drugs were dissolved at a concentration of 20 mg $ml^{-1}$ in dimethyl sulphoxide (DMSO). Each drug was diluted into DMSO before spreading on solid yeast media, such that varying amounts of drugs, but always equal volumes of DMSO (1% final), were added to each plate.

### *Yeast strains*

Yeast strains GA24 (MAT*a ura3 Gal$^+$ his3 barl suc2Δ9 pep4-3*), GA34 (formerly RS191 from R Sternglanz; MAT*a, ade2 ura3-1 his3-11 trpl-1 leu2-3, 2-112 top2-1 ts*), GA81 (formerly DY36 from T Hsieh; MAT *α/a ura3/ura3 trpl-1/trpl-1 his3-11,15/his3-11,15 leu2-3,112/leu2-3,112 adel/ADE1 lys2/LYS2 arg4-17/ARG4 TOP2/top2::LEU2* cir+), GA111 (formerly 362a from J Nitiss; *MATa adel ura3 leu2 ISE2 tyrl*), and GA199 (formerly DY25 from T Hsieh; MAT *α/a ura3/ura3 trpl-1/trpl-1 his3-11,15/his3-11,15 leu2-3,112/leu2-3,112 adel/ ADE1 lys2/LYS2 arg4-17/ARG4-17* cir+) were used in the present work. Media were prepared as described in Rose *et al.* (1990). Rich media [1% yeast extract, 2% glucose, 2% bactopeptone (Difco), 50 mg $l^{-1}$ adenine] is termed YPD; synthetic medium [0.67% yeast nitrogen base (Difco), 2% glucose, with added amino acids, adenine and uracil, where necessary] is called SD. Synthetic raffinose medium contains 2% raffinose and synthetic galactose medium contains 2% galactose, both instead of glucose. When cells were cultured in liquid medium, precultures were grown in 1% lactate/2% glycerol before adding 2% galactose, to avoid glucose inhibition of the *GAL1* UAS.

### *Plasmids*

Yeast topoisomerase II C-terminal truncations were constructed as follows: pGa1Top2Δ750 was constructed by deletion of the 2.5 kb *NheI–NheI* fragment from the pGa1Top2 plasmid (Worland and Wang, 1989); pGa1Top2Δ951 was constructed from pGa1Top2 by insertion of a stop codon at aa 951; pGa1Top2Δ1044 was constructed by deletion of the 1.4 kb *AvrII–NheI* fragment from the pGa1Top2 plasmid; pGa1Top2Δ1236 was constructed from pGa1Top2 by insertion of a stop codon at aa 1236 (see Figure 2). PGa1Top2Y783F plasmid was a kind gift of Dr UK Laemmli and contains a replacement of the active site tyrosine with phenylalanine. The purified mutant protein is completely inactive and cannot complement a *top2* mutant (data not shown). The construct expressing the C-terminus of the enzyme was constructed by digestion of the pGa1Top2 with *AgeI* and *PflMI* and blunt end ligation of the filled-in *AgeI* end with the trimmed *PflMI* end. It contains the first 4 aa of yeast topoisomerase II and its C-terminal domain from aa 1068 to aa 1429.

### *Viability assays*

Viability of yeast strains harbouring various plasmids was tested by plating 10 μl of appropriate medium containing either 400 or $10^3$ yeast cells onto agar plates of different growth media containing varying amounts of VP-16 drug dissolved in DMSO as indicated. In most cases 4-fold serial dilutions of the cells starting at 400 cells per 10 μl were plated for each concentration of VP-16. The control plates contained DMSO only. The agar plates were generally incubated at 30°C for 3 days.

### *Temperature-sensitive top2 complementation assay*

Yeast *top2* truncation mutants as well as the control plasmid (Yep24) and a plasmid pGa1Top2 bearing a full-length *TOP2* gene were transformed into GA34, which carries a *top2-1$^{ts}$* mutation (Dinardo *et al.*, 1984). Transformants were grown in SD media lacking uracil and then were streaked onto selective medium containing either glucose, raffinose or galactose. Growth was monitored at 25°C, 30°C or 36°C, as specified for 2–3 days.

### *Immunofluorescence*

Immunofluorescence of paraformaldehyde-fixed yeast spheroplasts was carried out as described elsewhere (Klein *et al.*, 1992; Palladino *et al.*, 1993), except that affinity-purified rabbit antibodies raised against full-length yeast topoisomerase II were used. Slides were mounted with 50% glycerol in phosphate-buffered saline (PBS) and 2 μg $ml^{-1}$ DAPI (4′6-diamidino-2-phenylindol-dihydrochloride) and photographed on a Zeiss Axiophot microscope using a 100x Pan Neofluar objective.

### *Protein extracts and Western blots*

Total protein extracts were obtained from the *pep4-3* mutant strain GA24 transformed with the plasmids carrying either the wild-type or mutant forms of *TOP2* downstream of the *GALI* UAS. Cultures were grown in glucose medium before being transferred to glycerol/lactate. Induction was for 2 h in the presence of 2% galactose and samples were taken from all three growth conditions. Cells were washed and frozen in liquid nitrogen before being lysed by vortexing with glass beads in a buffer containing 50 mM Tris-HCl pH 7.7, and protease and phosphatase inhibitors (Cardenas *et al.*, 1992). Aliquots were run on SDS–PAGE to determine relative protein concentrations and equal amounts of total protein from each condition were analysed by immunoblotting with affinity-purified antibodies directed against epitopes in the N-terminus of topoisomerase II. The peroxidase-coupled secondary antibody signal was visualised by enhanced chemiluminescence (ECL, Amersham).

### *Growth curves*

Growth curves were measured by dilution of overnight cultures to $5 \times 10^5$ cells $ml^{-1}$ in SD-uracil and manual counting of cells at hourly intervals in duplicate. Doubling time was calculated in the most rapid phase of growth, usually between 3 and 12 h after dilution into fresh medium.

### Results

The budding yeast, *S. cerevisiae*, has been used extensively as a test system for mutations that confer resistance to anti-tumour drugs that target topoisomerase II, but generally in screens for recessive mutations that leave the topoisomerase II active, but drug-resistant (Jannatipour *et al.*, 1993; Wasserman and Wang, 1994*a,b*; Liu and D'Arpa, 1992; Nitiss *et al.*, 1992). We have chosen to screen for forms of topoisomerase II that confer a dominant resistance phenotype like that reported in transfected human cells (Gudkov *et al.*, 1993), to eliminate the restriction that the drug-resistant form of topoisomerase II be enzymatically active.

### *Sensitivity of yeast to VP-16 depends on the culture media*

To optimise the yeast system for this analysis we have screened different yeast strains under different media

**Figure 1** Effect of culture medium on drug resistance. Four yeast strains (GA24, GA81, GA111 and GA199; see Materials and methods) were grown overnight to a density of $10^7$ cells $ml^{-1}$, and were diluted to $4\times10^4$ cells $ml^{-1}$ in the culture medium. Four hundred cells (10 $\mu$l) from each strain were plated onto a series of plates containing increasing concentrations of VP-16 (0, 2, 6, 20, 50 and 100 $\mu$g $ml^{-1}$) on a rich (YPD, **a**) or complete synthetic medium (SD, **b**). For **c**, two strains, GA24 and GA81 were transformed either with the Yep24 vector (multi-copy *URA3* plasmid) or with the same plasmid carrying the *GALl-TOP2* construct (here labelled pTOP2, Worland and Wang, 1989). Cells were grown overnight in SD-uracil and 400 cells were plated on selective medium (SD-uracil) containing a titration of the VP-16 drug (0, 1, 3, 10, 30 and 100 $\mu$g $ml^{-1}$). After 3 days at 30°C, the plates were photographed. All plates contain 1% DMSO final concentration.

conditions to assess their sensitivity to the anti-tumour drug, VP-16. These strains include GA24 and GA199, haploid and diploid strains respectively, that are wild-type for *TOP2*; GA81, a diploid strain carrying one disrupted copy of *TOP2*; and GA111, a strain carrying the *ISE2* mutation that was reported to enhance permeability to *m*-AMSA (Nitiss and Wang, 1988). Standardised plating assays show only slight variation among strains in their sensitivity to VP-16, with the *ISE2* strain showing no more sensitivity than the other strains tested. We find, however, that all strains are more sensitive to VP-16 on synthetic (SD), as compared with rich (YPD) medium (Figure 1a for rich media, and 1b for SD or synthetic media). This may reflect media-dependent alterations in plasma membrane permeability or changes in drug metabolism within the cell. The plating of cells in osmotically stabilised media (with 1M sorbitol) after enzymatic removal of the cell wall, did not alter the lack of drug sensitivity on YPD, thus the cell wall does not limit drug accessibility (data not shown).

Our system for ectopic expression of topoisomerase II in yeast requires growth under selective conditions (SD lacking uracil). If our test strain, GA24, carries only the vector (Yep24), cells are sensitive to as little as 30 $\mu$g $ml^{-1}$ VP-16, and no growth is observed at either 50 $\mu$g $ml^{-1}$ (not shown) or 100 $\mu$g $ml^{-1}$ (Figure 1c). When the Yep24 vector carries the full length *TOP2* gene, we see growth inhibition at 10 $\mu$g $ml^{-1}$ VP-16 instead of 30 or 100 $\mu$g $ml^{-1}$ (Figure 1c). The plasmid-borne *TOP2* gene is expressed at a low level under these conditions, but nonetheless renders the cell slightly more sensitive to the inhibitor. This is consistent with previous papers reporting that increased levels of topoisomerase II correlate with the cell's sensitivity to VP-16, VM-26 and other topoisomerase II targeting reagents (Nitiss *et al.*, 1992; Eder *et al.*, 1993; and reviewed in Beck *et al.*, 1993; Corbett and Osheroff, 1993; Liu and D'Arpa, 1992; Alton and Harris, 1993). In the case of GA81, which is a diploid heterozygotic for the *TOP2* locus, we observe somewhat better growth in the presence of VP-16, perhaps reflecting an overall lower level of enzyme. For the sake of standardisation, most experiments will be presented using GA24, although similar results were also obtained with GA81.

### *Characterisation of topoisomerase II mutants expressed* in vivo, *and their effect on viability*

The above results show that yeast carrying either a vector alone or the vector with the *TOP2* gene are sensitive to VP-16 at concentrations between 10 to 30 $\mu$g $ml^{-1}$. In order to analyse the effect of mutant forms of topoisomerase II on drug sensitivity, we checked whether strains expressing the plasmid-borne forms of topoisomerase II were viable in the absence of drugs, whether the truncated forms complement a temperature-sensitive *top2* mutation, and, if so, at which levels of expression. The constructs used are C-terminal truncations, of which all but the shortest leave the active site of the enzyme intact and retain the conserved regions homologous to type II topoisomerases, including GyrA and GyrB of *E. coli* (see Figure 2). The truncations remove the major sites of casein kinase II modification in the C-terminal 200 aa of the enzyme (indicated as arrows above the gene), and in all but the truncation at aa 1236, nuclear localisation signals (between aa 1166 and 1208, Caron *et al.*, 1994) are removed. All constructs are inserted in the same multicopy vector with a galactose-inducible promoter, which allows differential levels of transcription depending on the carbon source, i.e. glucose allows low levels; raffinose or glycerol/lactate, intermediate; and galactose, high levels of expression.

Although the *GALI* UAS is repressed on glucose, sufficient topoisomerase II is synthesised to allow growth in a *top2-1* temperature-sensitive mutant at non-permissive temperature (see Table I, 36°C). We similarly screened for the ability of the truncated forms to complement the *top2-1* temperature-sensitive mutant at non-permissive temperature (36°C) on different carbon sources. None of the shorter truncations (pGal1Top2Δ1044, Δ951 or Δ750) is able to maintain mitotic growth at any level of expression (Table I), suggesting that these are inactive forms of the enzyme. The truncation at aa 1236 (Δ1236) complements better on raffinose than on glucose, suggesting that its level of expression must be elevated to provide sufficient topoisomerase II activity for mitotic growth (Table I). Galactose-induced expression of either the full-length *TOP2* or the Δ1236 gene from the multicopy vector is lethal both for wild-type yeast cells (data not shown) and for the *top2-1* ts mutant at permissive temperature (see Table I, see galactose, 25°C and 30°C). In contrast, none of the shorter C-terminally truncated forms of topoisomerase II is detrimental to growth at any level of expression (see Table I).

The inability of the shorter topoisomerase II proteins to inhibit growth when overexpressed, or to complement *top2-1* at low levels of expression could simply reflect instability of the message or protein. To check this we have probed for the presence of the truncated gene products, as well as the wild-type protein and an active site mutant (Y783F) in whole cell extracts of GA24 transformed with the appropriate plasmids and grown in galactose. By Western blotting equal amounts of whole cell extract from each indicated transformant, we readily detect all the mutated forms of topoisomerase II (Figure 3c). For comparison of these protein amounts with endogenous levels of full-length enzyme, a longer exposure of the blot of galactose grown cells is shown (Figure 3d). In all cases the correct-sized proteins are present, and on galactose all are expressed and stable at levels much greater than the endogenous protein. The truncations are below the level of detection under standard blotting conditions in cells grown on either glycerol/lactate or on glucose media (Figure 3a and b).

In conclusion, the full-length *TOP2* construct and the truncation at Δ1236 can complement a *top2-1^ts* mutant on the multicopy vector in glucose-containing media, and at this level of expression no construct is detrimental to growth. The truncations at Δ1044, Δ951 and Δ750 cannot complement a *top2-1^ts* mutant, although the proteins are synthesised and are relatively stable. Since our system does not require that the plasmid-borne topoisomerase II complement a *top2* mutant, we could screen for drug resistance conferred by the plasmid-borne constructs on glucose, and thus avoid any toxicity owing to high level expression of the polypeptides.

**Figure 2** Construction of the C-terminal truncations of the yeast *TOP2* gene. The truncations were constructed as follows: *Δ*750 was constructed by deletion of the 2.5 kb *Nhe*I–*Nhe*I fragment from the pGalTop2 plasmid (Worland and Wang, 1989). The last amino acid before the stop that is native to yeast topoisomerase II is aa 750, hence its name. Δ951 was constructed from the pGalTop2 by insertion of a stop codon at aa 951. Δ1044 was constructed by deletion of the 1.4 kb *Avr*II–NheI fragment from the pGalTop2 plasmid; the last amino acid native to yeast topoisomerase II is aa 1044. Δ1236 was constructed from pGalTop2 by insertion of a stop codon at aa 1236. All truncations were constructed in the host plasmid pGalTop2 (Worland and Wang, 1989). The arrows above the full-length topoisomerase II map indicate putative sites of casein kinase II phosphorylation, whereas NLS indicates the proposed location of the nuclear localisation signal (Shiozaki and Yanagida, 1991; Caron *et al.*, 1994; Crenshaw and Hsieh, 1993*a*, *b*). Alignment with the prokaryotic type II topoisomerase (gyrase) is indicated below the yeast enzyme.

**Table I** A screen for viability of the *top2-1$^{ts}$* strain carrying the various *TOP2* alleles on the multi-copy plasmid pGalTop2 (see Figure 2) at permissive (25°C), semi-permissive (30°C) and non-permissive temperatures (36°C)

| *Construct* | *Glucose* 25° | *Glucose* 30° | *Glucose* 36° | *Raffinose* 25° | *Raffinose* 30° | *Raffinose* 36° | *Galactose* 25° | *Galactose* 30° | *Galactose* 36° |
|---|---|---|---|---|---|---|---|---|---|
| Yep24-no topoII | + | + | – | + | + | – | + | + | – |
| YepGalTop2 Δ750 | + | + | – | + | + | – | + | + | – |
| YepGalTop2 Δ951 | + | + | – | + | + | – | + | + | – |
| YepGalTop2 Δ1044 | + | + | – | + | + | – | + | + | – |
| YepGalTop2 Δ1236 | + | +/– | –/+ | + | + | + | +/– | – | – |
| YepGalTop2 | + | + | + | + | + | + | – | – | – |

All transformants were grown on synthetic medium lacking uracil, but the carbon source (indicated above the growth temperature) was varied to modulate expression levels. The mutant genes are under control of a *GAL1* UAS, and expression levels are highest on galactose, intermediate on raffinose and low on glucose. Normal growth is indicated by +, no growth by –, and slow growth by +/– or –/+.

### *Truncated forms of topoisomerase II can confer a dominant drug resistance phenotype*

We have plated serial dilutions of GA24 cells transformed with either the truncated or wild-type *TOP2* alleles in the pGalTop2 vector onto media containing increasing amounts of VP-16. Transformants of GA24 (Figure 4) carrying wild-type *TOP2* grow poorly, or not at all, on plates containing 20 $\mu$g ml$^{-1}$ VP-16. Resistance to up to 40 $\mu$g ml$^{-1}$ VP-16 is conferred by the presence of truncated constructs pGalTop2Δ951, pGalTop2Δ1044 and pGalTop2Δ1236, while strains carrying pGalTop2Δ750 resemble those carrying the wild-type gene (Figure 4). In the diploid GA81, resistance of the same constructs was observed at 80 $\mu$g ml$^{-1}$ VP-16 (Figure 5). Finally, a very similar pattern of slightly elevated resistance was observed when etoposide was replaced by the related topoisomerase II-inhibitor, teniposide (VM-26, data not shown), consistent with the idea that these two agents interact with the enzyme in a similar manner. In all cases the resistance to elevated drug levels is reproducible and construct-dependent.

To see if the C-terminus of topoisomerase II alone can influence drug resistance, the first 4 aa of topoisomerase II were fused to the last 361 aa, and the construct was introduced into the two test strains GA24 and GA81. The C-terminus of topoisomerase II is not highly stable, but can be detected by Western blot (data not shown). The level of expression attained has no effect on growth rate in the absence of drug, nor does it confer drug resistance (data not shown). Assuming that the larger fragments of topoisomerase II confer drug resistance by binding the drug, but not damaging DNA, our results suggest that the C-terminal domain of topoisomerase II is not necessary for binding epipodophyllotoxins, nor are the first 750 aa sufficient for efficient interaction.

### *The active site mutant Y783F confers a dominant drug resistance phenotype when expressed ectopically*

Two phenomena might account for the resistance to an elevated level of topoisomerase II inhibitors conferred by the Δ1044 construct, which lacks a dimerisation domain, the nuclear localisation signal (NLS), and fails to complement *top2* deficiency *in vivo*. First, if the Δ1044 protein is primarily

**Figure 3** Detection of the truncated topoisomerase II proteins by Western blot. GA24 yeast cells were transformed with the truncation mutants indicated in Figure 2, the Y783F active site mutant or the wild-type *TOP2* gene, all carried on the pGalTop2 plasmid. Cells grown on different media at 30°C were lysed and whole-cell extracts were analysed by 7.5% SDS–PAGE. Equal amounts of protein from each transformant were transferred to a nitrocellulose filter and probed with anti-topoisomerase II N-terminal antibodies, which were visualised by enhanced chemiluminescence (ECL, Amersham). (**a**) The indicated transformants were grown in glucose medium. (**b**) The same strains were grown in glycerol/lactate medium. (**c**) Cells grown in glycerol/lactate medium supplemented with 2% galactose for 2 h before lysis. (**d**) A longer exposure of the gel shown in (**c**) so that the full-length topoisomerase II can be seen (arrow). The spot in (**a**) indicates a proteolytic product that cross-reacts with the antibody. The smaller bands seen upon galactose induction are consistent with the sizes for truncated forms of topoisomerase II expected from the constructs. Molecular weight markers are given as kDa.

**Figure 4** Drug resistance of GA24 transformed with truncated forms of DNA topoisomerase II. The GA24 strain was transformed with plasmids containing the full-length *TOP2* gene (pGalTop2, labelled WT), different C-terminal truncations of yeast topoisomerase II (sites of truncation indicated by Δ750, Δ951, Δ1044 and Δ1236). Equal numbers of cells in a dilution series (400, 100 and 25 cells always in 10 $\mu$l) were plated on minimal selective medium containing increasing concentrations of etoposide (VP-16, as indicated). Growth was for 3 days at 30°C.

cytoplasmic, as expected from its lack of NLS, it may bind the drug before it has access to the nuclear-localised, full-length topoisomerase II. Alternatively, having an inactive complement of enzyme may simply reduce the damage induced by the drug by not forming the 'cleavable complex' in which the enzyme is covalently bound to DNA. To see whether catalytic inactivity is sufficient to confer drug resistance, we tested the plasmid-borne active site mutant (Y783F) in an identical assay.

As shown in Figure 6, low-level expression of the active site mutant (pGalTop2Y783F) is sufficient to confer drug resistance at concentrations up to 80 $\mu$g ml$^{-1}$ etoposide. In repeated assays the resistance conferred by the active site mutant is reproducibly higher than that conferred by the truncated forms of topoisomerase II (Δ1236 and Δ1044; ER and YSV, data not shown). Slight variations observed in the level of drug tolerated by the transformants are likely to reflect varying plasmid and expression levels in different cells.

### *Mutation of CKII phosphorylation sites does not alter drug sensitivity*

We have previously identified casein kinase II as the major kinase modifying yeast topoisomerase II *in vivo*, and have

**Figure 5** Drug resistance of GA81 transformed with truncated forms of DNA topoisomerase II. Roughly $10^3$ cells of the diploid yeast strain GA81 transformed either with the truncation mutants pGalTop2Δ750, pGalTop2Δ951, pGalTop2Δ1044 or pGalTop2Δ1236 or with pGalTop2 carrying wild-type *TOP2* (WT) or the Yep24 plasmid alone (control) were plated on selective medium containing increasing amounts of VP-16 (0, 8, 25 and 80 $\mu$g ml$^{-1}$) and grown for 3 days at 30°C.

**Figure 6** Drug resistance of GA24 transformed with the active site mutant and casein kinase II target site mutants of DNA topoisomerase II. The GA24 strain was transformed with plasmids containing the wild-type full-length *TOP2* gene (labelled WT), the full-length *TOP2* gene with a Y783F mutation at the active site (labelled Y783F) or the full-length *TOP2* gene with the point mutations indicated above the linear map of yeast *TOP2*, all of which mutate potential casein kinase II acceptor sites from the phosphate acceptor residue to a non-charged non-acceptor amino acid. The mutated sites are clustered in three groups representing tryptic peptides numbered 6, 8 and 9; as indicated in the map of topoisomerase II and defined in Cardenas *et al.* (1992). Equal numbers of cells in serial dilutions (400, 100 and 25 cells) were plated on minimal selective medium (SD-uracil) containing increasing concentrations of etoposide ($\mu$g VP-16 ml$^{-1}$, as indicated). Growth was for 3 days at 30°C.

mapped the sites of modification to the C-terminal domain (Cardenas *et al.*, 1992; Dang *et al.*, 1994; Alghisi *et al.*, 1994). The putative acceptor sites are found clustered together on a limited number of tryptic peptides, which allowed us to create three groups of point mutations that eliminate the target sites on three different peptides (see map, Figure 6; Cardenas *et al.*, 1992; Alghisi *et al.*, 1994). The three series of clustered mutations sites (replacement of Ser or Thr by Ala or Gly), were at aa 1087 and aa 1088 (labelled 6); aa 1267, aa 1270 and aa 1273, (labelled 8); and aa 1357, aa 1364, and aa 1366 (labelled 9). By substitution of acceptor sites, the mutations mimic an under-phosphorylated form of topoisomerase. In contrast to the active site mutant, these plasmids carrying point mutations in the C-terminus of topoisomerase II do not significantly alter the sensitivity of the host cell to VP-16 (Figure 6).

### *Are the resistance-conferring forms of topoisomerase II cytosolic?*

The active site mutant and the two truncations, Δ951 and Δ1044, are non-complementing activities that confer drug resistance. The longer truncated form of topoisomerase II (Δ1236) is active, but unlike the hypersensitivity conferred by expression of the full-length protein, expression of Δ1236 confers drug resistance. Is this because it is less efficiently localised to the nucleus (e.g. Crenshaw and Hsieh, 1993*b*)? To check the intracellular localisation of the mutant forms of topoisomerase II, we have induced the topoisomerase II constructs for a limited time on galactose and used indirect immunofluorescence to reveal the distribution of the enzyme in paraformaldehyde-fixed cells. Figure 7 shows the distribution of the mutant and wild-type *TOP2* proteins, as detected by indirect immunofluorescence. Because our antibodies cannot distinguish between truncated and wild-type topoisomerase II forms, we always detect low-level immunofluorescence of the endogenous, exclusively nuclear wild-type enzyme (see Figure 7a; Klein *et al.*, 1992).

The deletion of the C-terminal 193 aa (Δ1236) leads to the appearance of significant cytoplasmic staining in addition to strong nuclear staining, while deletion of 385 aa (Δ1044) leads to a strong cytoplasmic staining with weak nuclear staining (Figure 7c and d). Cytoplasmic staining is also observed in the shorter truncations Δ951 and Δ750, although in these cases the endogenous, wild-type topoisomerase II gives a more prominent signal, because the truncated forms contain fewer epitopes recognised by the polyclonal antibody. As expected, in the strain carrying the pGalTop2Y783F active site mutant, we observe exclusively nuclear staining. The negative control with only secondary antibodies is shown in Figure 7g, confirming that the cytosolic staining in Figure 7b–e is not due to the secondary antibody. Similar results of protein distribution were obtained in crude fractionations of the yeast cell into cytoplasmic and nuclear fractions using sucrose gradients (data not shown).

### *Ectopic expression of truncated forms of topoisomerase II do not interfere with growth rates*

The enhanced drug resistance observed in some transformants might be attributed to an altered cell cycle that could either allow degradation of the drug or longer repair times after damage is incurred. To check for alteration in the growth pattern of the transformants, growth curves and doubling times were calculated for the strains carrying truncated forms of topoisomerase II. All have essentially identical growth curves (Figure 8), with only the strain carrying the Δ951 mutant showing a slightly longer doubling time (156 min doubling time, as opposed to 139 min or 140 min for the wild-type or Δ1044 transformant respectively). Thus, we see no consistent correlation between the division time of these strains and their drug resistance, suggesting again that the resistance phenotype probably results directly from expression of the non-complementing topoisomerase II domains.

### Discussion

Drug resistance due to alterations in DNA topoisomerase II levels or structure occurs frequently in situations of high-dose chemotherapy (Beck *et al.*, 1993; Corbett and Osheroff, 1993). It is essential to understand the mechanisms by which cells become resistant to topoisomerase II inhibitors to be able to counter this tendency and increase the chances of successful anti-tumour treatment. Three kinds of experimental systems are used to study the phenomenon of topoisomerase II-related drug resistance. The first approach is based on the isolation and characterisation of drug-resistant cancer cells from patients or laboratory animals (Long *et al.*, 1991; Danks *et al.*, 1993). The second consists of

**Figure 7** Analysis of subcellular localisation of the truncated topoisomerase II. Immunofluorescence to topoisomerase II is shown for GA24 yeast cells carrying the uninduced wild-type *TOP2* gene (WT, **a**, **g**) or transformed with the truncation mutants as indicated in the pGALTOP2 vector (**b**–**f**). Transcription was induced by the addition of galactose for 2 h at 30°C (for pGalTop2Δ1044, pGalTop2Δ1236 and pGalTop2Y783F; and for 6 h in the case of pGalTop2Δ750 and pGalTop2Δ951). Cells were spheroplasted, fixed and probed with affinity-purified anti-topoisomerase II and a FITC-conjugated secondary antibody. DNA visualised by DAPI is shown on the left and the FITC signals from the anti-topoisomerase II reaction on the same cells to the right. The background staining contributed by the secondary antibody alone is shown in **g**. Bar = 4 μm.

**Figure 8** Growth curves of GA24 transformed with a multicopy vector carrying mutant and wild-type *TOP2* alleles. GA24 yeast cells transformed with either the wild-type pGalTop2 plasmid, or with the indicated truncation mutants on pGalTop2, were grown overnight in a SD-uracil liquid medium to a density of *c* $2 \times 10^7$ cells $ml^{-1}$ then diluted to a density of $5 \times 10^5$ cells $ml^{-1}$ into fresh medium and grown at 30°C. Samples were taken at the indicated times, immediately fixed by addition of formaldehyde to a final concentration of 3.7%, and counted manually in duplicate. The data represent the results of two independent experiments.

selection *in vitro* of cultured cancer cells resistant to topoisomerase II-targeted drugs (McPherson *et al.*, 1993; Chan *et al.*, 1993), and the third approach uses simple and genetically accessible experimental systems like yeast to analyse the phenomenon of drug resistance (Wasserman and Wang, 1994*a*, *b*; Nitiss and Wang, 1988; Jannatipour *et al.*, 1993; Liu *et al.*, 1994). To obtain drug-resistant strains of yeast, several groups have mutagenised the gene for topoisomerase II *in vitro* and assessed the effect of the mutant gene on drug resistance in a temperature-sensitive yeast strain (Wasserman and Wang, 1994*a*; Liu *et al.*, 1994). A variation of this approach involves the transfection by a retroviral vector containing an expression library of topoisomerase IIα subfragments in human cultured cells and the subsequent selection of stable drug-resistant transformants (Gudkov *et al.*, 1993). These workers found that the majority of the resistance-conferring clones encoded antisense RNA that lowered cellular levels of topoisomerase IIα. However, three sense clones apparently allowed the synthesis of fragments of the enzyme which were able to enhance resistance to VP-16 and/or *m*-AMSA by 3- to 5-fold. No immunolocalisation or Western blotting was done to confirm this interpretation, but if correct, it might provide a means to screen for minimal drug binding sites within topoisomerase II.

We have adapted this novel approach and applied it to the study of drug resistance in yeast, by screening for a drug resistance phenotype mediated *in trans* by plasmid-borne forms of topoisomerase II in strains that contain a wild-type DNA topoisomerase II enzyme. Our system thus reflects the situation in mammalian cells, where mutations incurred in one of the two alleles confer a dominant drug resistance phenotype, while the wild-type allele provides the essential enzymatic activity.

We find that the low level expression of truncated forms of yeast topoisomerase II leads to increased survival in the presence of VP-16 and VM-26, while ectopic expression of the wild-type allele confers enhanced sensitivity to these antitumour drugs. The most efficient drug resistance is mediated by forms of topoisomerase II that are unable to complement a *top2-1*$^{ts}$ mutant. A smaller truncation that removes the active site (Δ750) confers little or no resistance, suggesting that the N-terminal 750 aa are not sufficient for this phenotype. Importantly, two truncations that confer drug resistance (Δ1044, Δ951) are not detrimental to the growth rate of cells and cannot complement a lethal *top2* mutation, even when overexpressed. We demonstrate that these forms are stable and are largely localised to the cytoplasm (Figure 6), suggesting that they may act as a cytosolic sink for the drug, diminishing the level of etoposide that reaches the nuclear topoisomerase II. An alternative explanation is that the mutant forms have an allosteric effect on the wild-type topoisomerase II, which could reduce either the enzyme's accessibility or the effects of VP-16 binding. However, one would expect such allosteric effects to interfere with topoisomerase II function and cause a growth defect, particularly when the truncated form is overexpressed. This was not observed (Table I; Figure 8).

A second pattern of drug resistance is mediated by the active site mutation (Y783F). The Y783F mutant form of topoisomerase II is catalytically inactive and non-complementing (G-C Alghisi, unpublished results), but in contrast to Δ1044, it is entirely nuclear localised. The high level of drug resistance may either reflect preferential interaction of the drug with the mutated enzyme that can no longer damage the genomic DNA, or the presence of heterodimers (formed from one wild-type and one mutant subunit) that effectively reduces the active population of topoisomerase II. Drug resistance in this latter case would be similar to many instances observed in drug-resistant mammalian cell lines with lowered levels of active topoisomerase II (reviewed in Beck *et al*., 1993). Further study of differentially tagged forms and purification of enzyme may be required to fully explain the mechanism of the drug-resistance conferred by expression of an inactive form of topoisomerase II in yeast.

Two previous reports also found truncated forms of topoisomerase II that confer dominant drug resistance, although the presence of secondary point mutations was not excluded. In one case a murine topoisomerase IIα, truncated at aa 1148, confers resistance to adriamycin in the presence of the wild-type topoisomerase II allele (McPherson *et al*., 1993), as does an unidentified truncation that produces a 160 kDa form of topoisomerase II with altered subcellular localisation (Feldhoff *et al*., 1994). In yeast two truncated forms, terminating at aa 1235 and aa 1191, respectively, were shown to confer a low level of drug resistance to amsacrine (at 10 $\mu g\ ml^{-1}$; Wasserman and Wang, 1994*a*). In contrast to our conditions, these mutant forms were selected for their ability to support growth as well as confer drug resistance, hence they were at least partially nuclear and enzymatically active (Wasserman and Wang, 1994*a*; Caron *et al*., 1994). Our screen for dominant drug resistance in yeast can be readily applied to domains of the human topoisomerase IIα gene, since we are not dependent on complementation of the *top2$^{ts}$* deficiency.

A final class of mutation that correlates with drug resistance involves the phosphorylation level of topoisomerase II. It is known that phosphorylation of topoisomerase II increases its activity *in vitro*, while dephosphorylation lowers enzymatic activity (reviewed in Cardenas and Gasser, 1993). Consistently, an amsacrine-resistant cell line was reported to have a 2- to 3-fold drop in the population of newly synthesised, phosphorylated topoisomerase IIα (Ganapathi *et al*., 1993). On the other hand, a hyperphosphorylated form of topoisomerase II was also reported in an etoposide-resistant human cancer cell line (Takano *et al*., 1991). Interestingly, in this case hyperphosphorylation coincided with a 10-fold decrease in the topoisomerase II protein level, which effectively lowered the concentration of drug target. Osheroff and colleagues have demonstrated that phosphorylation by either CKII or PKC *in vitro* renders topoisomerase II less sensitive to inhibition by VP-16 or VM-26 (DeVore *et al*., 1992), thus suggesting that a hyperphosphorylated form of topoisomerase II might correlate with drug resistance *in vivo*, while cells bearing an under-phosphorylated form might be less resistant. We have tested point mutations that significantly reduce the number of CKII target sites on topoisomerase II (Alghisi *et al*., 1994), and find no significant variation in drug resistance. This lack of increased sensitivity (or enhanced resistance) suggests that the previously reported correlation between drug resistance and the phosphorylation state of topoisomerase II is either invalid for yeast, reflects phosphorylation by another kinase, or is due to other parameters, such as lowered expression levels of the enzyme (Takano *et al*., 1991; Ganapathi *et al*., 1993). We also observe that elimination of the C-terminal domain, which contains targets for other kinases as well as CKII, improves, rather than reduces, drug resistance in yeast.

The level of drug resistance we observe in yeast is only 2- to 4-fold over the sensitivity of a wild-type strain. This appears modest when compared to the drug resistance in tumour cell lines, which can grow in 20- to 50-fold the amount of drug tolerated by normal cells (reviewed in Beck *et al*., 1993). Drug-resistant tumour cells usually incur multiple mutations, however, and the resistance is an accumulative effect of mutations in *MDR1*, topoisomerase IIα and perhaps other unknown targets of mutation. Our degree of enhanced resistance is comparable to the levels observed in transfected HeLa cells, in which small subfragments of human *Top2α* gene were randomly expressed (Gudkov *et al*., 1993). In this HeLa cell study, it was not determined whether the cloned fragment made a stable polypeptide product, nor was the subcellular localisation of the products examined. Indeed, the predicted polypeptide products were small enough to readily diffuse through nuclear pores ($<20$ kDa), hence the observed effect might have been due to an allosteric down-regulation of the endogenous topoisomerase II activity. Possible alterations in the growth rates of the drug-resistant cells were not reported.

Our study here has clearly established the fact that a low-level expression of inactive forms of DNA topoisomerase II can confer a certain level of drug resistance on otherwise wild-type yeast cells, i.e. cells with a wild-type genomic allele of *TOP2*. This is clinically relevant, since at early stages in the development of drug resistance, a single mutation in one of the alleles encoding topoisomerase IIα may initially confer a growth advantage on a subset of tumour cells. These cells are then likely to incur further mutations that confer yet higher levels of drug resistance. One immediate application of this assay for a plasmid-mediated drug resistance will be to define protein domains that bind topoisomerase II-inhibiting drugs with high efficiency. Understanding both the mode and site of action for topoisomerase II-targeted drugs will be essential for the improvement of high-dose chemotherapy.

**Acknowledgements**
We would like to thank R Sternglanz, J Nitiss and T Hsieh for yeast strains, and UK Laemmli and JC Wang for plasmids. Research in the Gasser laboratory is supported by the Swiss Cancer League (Krebsforschung Schweiz), the Swiss National Science Foundation and the Human Frontiers Science Program. YSV thanks EMBO for an East European Fellowship.

### References

ALGHISI G-C, ROBERTS E, CARDENAS ME AND GASSER SM. (1994). The regulation of DNA topoisomerase II by casein kinase II. *Cell Mol. Biol. Res*., **40**, 563–572.

ALTON PA AND HARRIS AL. ((1993). The role of DNA topoisomerase II in drug resistance. *Br. J. Haematol*., **85**, 241–245.

BECK WT, DANKS MK, WOLVERTON JS, KIM R AND CHEN M. (1993). Drug resistance associated with altered DNA topoisomerase II. *Adv. Enz. Regulation*, **33**, 113–127.

BUGG BY, DANKS MK, BECK WT AND SUTTLE DP. (1991). Expression of a mutant DNA topoisomerase II in CCRF-CEM human leukemic cells selected for resistance to teniposide. *Proc. Natl Acad. Sci. USA*, **88**, 7654–7658.

CAMPAIN JA, GOTTESMAN MM AND PASTAN I. (1994). A novel mutant topoisomerase IIα present in VP-16-resistant melanoma cell lines has a deletion of alanine 429. *Biochemistry*, **33**, 11327–11332.

CARDENAS ME AND GASSER SM. (1993). Regulation of topoisomerase II by phosphorylation: a role for casein kinase II. *J. Cell. Sci*., **104**, 219–225.

CARDENAS ME, DANG Q, GLOVER CVC AND GASSER SM. (1992). Casein kinase II phosphorylates with eukaryotic-specific C-terminal domain of topoisomerase II *in vivo*. *EMBO J*., **11**, 1785–1796.

CARON PR, WATT P AND WANG JC. (1994). The C-terminal domain of *Saccharomyces cerevisiae* DNA topoisomerase II. *Mol. Cell Biol.*, **14**, 3197–3207.

CHAN VT, NG SW, EDER JP AND SCHNIPPER LE. (1993). Molecular cloning and identification of a point mutation in the topoisomerase II cDNA from an etoposide-resistant Chinese hamster ovary cell line. *J. Biol. Chem.*, **268**, 2160–2165.

CORBETT AH AND OSHEROFF T. (1993). When good enzymes go bad: conversion of topoisomerase II to a cellular toxin by antineoplastic drugs. *Chem. Res. Toxicol.*, **6**, 585–597.

CRENSHAW DG AND HSIEH T. (1993*a*). Function of the hydrophilic carboxyl terminus of type II DNA topoisomerase from *Drosophila melanogaster*. I. *in vitro* studies *J. Biol. Chem.*, **268**, 21328–21334.

CRENSHAW DG AND HSIEH T. (1993*b*). Function of the hydrophilic carboxyl terminus of type II DNA topoisomerase from *Drosophila melanogaster*. II. *In vivo* studies. *J. Biol. Chem.*, **268**, 21335–21343.

DANG O, ALGHISI G-C AND GASSER SM. (1994). Phosphorylation of the C-terminal domain of yeast topoisomerase II by casein kinase II affects DNA-protein interaction. *J. Mol. Biol.*, **243**, 10–24.

DANKS MK, WARMOTH MR, FRICHE E, GRANZEN B, BUGG BY, HARKER WG, ZWELLING LA, FUTSCHER BW, SUTTLE DP AND BECK WT. (1993). Single-strand conformational polymorphism analysis of the M(r) 170,000 isozyme of DNA topoisomerase II in human tumour cells. *Cancer Res.*, **53**, 1373–1379.

DEVORE RF, CORBETT AH AND OSHEROFF N. (1992). Phosphorylation of topoisomerase II by casein kinase II and protein kinase C: Effects on enzyme-mediated DNA cleavage/religation and sensitivity to the antineoplastic drugs etoposide and *m*-AMSA. *Cancer Res.*, **52**, 2156–2161.

DINARDO S, VOELKEL K AND STERNGLANZ R. (1984). DNA topoisomerase II mutant of *Saccharomyces cerevisiae*: topoisomerase II is required for segregation of daughter molecule at the termination of DNA replication. *Proc. Natl Acad. Sci. USA*, **82**, 2616–2620.

EDER JJ, CHAN VT, NIEMIERKO E, TEICHER BA AND SCHNIPPER LE. (1993). Conditional expression of wild-type topoisomerase II complements a mutant enzyme in mammalian cells. *J. Biol. Chem.*, **268**, 13844–13849.

ENDICOTT JA AND LING V. (1989). The biochemistry of P-glycoprotein-mediated multidrug resistance. *Annu. Rev. Biochem.*, **58**, 137–171.

FELDHOFF PW, MIRSKI SE, COLE SP AND SULLIVAN DM. (1994). Altered subcellular distribution of topoisomerase IIα in a drug-resistant human small cell lung cancer cell line. *Cancer Res.*, **54**, 756–762,

GANAPATHI R, ZWELLING L, CONSTANTINOU A, FORD J AND GRABOWSKI D. (1993). Altered phosphorylation, biosynthesis and degradation of the 170 kDa isoform of topoisomerase II in amsacrine-resistant human leukemia cells. *Biomed. Biophys. Res. Commun.*, **192**, 1274–1280.

GRANZEN B, GRAVES DE, BAGULEY BC, DANKS MK AND BECK WT. (1992). Structure-activity studies of amsacrine analogs in drug resistant human leukemia cell lines expressing either altered DNA topoisomerase II or P-glycoprotein. *Oncol. Res.*, **4**, 489–496.

GUDKOV AV, ZELNICK CR, KAZAROV AR, THIMMAPAYA R, SUTTLE DP, BECK WT AND RONINSON IB. (1993). Isolation of genetic suppressor elements, inducing resistance to topoisomerase II-interactive cytotoxic drugs, from human topoisomerase II cDNA. *Proc. Natl Acad. Sci. USA*, **90**, 3231–3235.

HINDS M, DEISSEROTH K, MAYES J, ALTSCHULER E, JANSEN R, LEDLEY FD AND ZWELLING LA. (1991). Identification of a point mutation in the topoisomerase II gene from a human leukemia cell line containing an amsacrine-resistant form of topoisomerase II. *Cancer Res.*, **51**, 4729–4731.

JANNATIPOUR M, LIU YX AND NITISS JL. (1993). The *top2-5* mutant of yeast topoisomerase II encodes an enzyme resistant to etoposide and amsacrine. *J. Biol. Chem.*, **268**, 18586–18592.

KLEIN F, LAROCHE T, CARDENAS ME, HOFMANN JF, SCHWEIZER D AND GASSER SM. (1992). Localization of RAP1 and topoisomerase II in nuclei and meiotic chromosomes of yeast. *J. Cell Biol.*, **117**, 935–948.

LEE MS, WANG JC AND BERAN M. (1992). Two independent amsacrine-resistant human myeloid leukemia cell lines share an identical point mutation in the 170 kDa form of human topoisomerase II. *J. Mol. Biol.*, **223**, 837–843.

LIU LF. (1989). DNA topoisomerase poisons as antitumour drugs. *Annu. Rev. Biochem.*, **58**, 351–375.

LIU LF AND D'ARPA P. (1992). Topoisomerase-targeting antitumour drugs: mechanisms of cytotoxicity and resistance. *Impt. Adv. in Oncol.*, 79–89.

LIU YX, HSIUNG Y, JANNATIPOUR M, YEH Y AND NITISS JL. (1994). Yeast topoisomerase II mutants resistant to anti-topoisomerase agents: identification and characterization of new yeast topoisomerase II mutants selected for resistance to etoposide. *Cancer Res.*, **54**, 2943–2951.

LONG BH, WANG L, LORICO A, WANG RC, BRATTAIN MG AND CASAZZA AM. (1991). Mechanisms of resistance to etoposide and teniposide in acquired resistant human colon and lung carcinoma cell lines. *Cancer Res.*, **51**, 5275–5283.

MCPHERSON JP, BROWN GA AND GOLDENBERG GJ. (1993). Characterization of a DNA topoisomerase IIα gene rearrangement in adriamycin-resistant P388 leukemia: expression of a fusion messenger RNA transcript encoding topoisomerase IIα and the retinoic acid receptor alpha locus. *Cancer Res.*, **53**, 5885–5889.

NITISS J AND WANG JC. (1988). DNA topoisomerase-targeting antitumour drugs can be studied in yeast. *Proc. Natl Acad. Sci. USA*, **85**, 7501–7505.

NITISS JL, LIU YX, HARBURY P, JANNATIPOUR M, WASSERMAN RA AND WANG JC. (1992). Amsacrine and etoposide hypersensitivity of yeast cells overexpressing DNA topoisomerase II. *Cancer Res.*, **52**, 4467–4472.

PALLADINO F, LAROCHE T, GILSON E, AXELROD A, PILLUS L AND GASSER SM. (1993). SIR3 and SIR4 proteins are required for the positioning and integrity of yeast telomeres. *Cell*, **75**, 543–555.

POMMIER Y. (1993). DNA topoisomerase I and II in cancer therapy: update and perspectives. *Cancer Chemother. Pharmacol.*, **3**, 103–108.

ROSE MD, WINSTON F AND HEITER P. (1990). *Methods in Yeast Genetics. A Laboratory Course Manual*, pp. 198, Cold Spring Harbor Laboratory Press: Cold Spring Harbor, New York.

SHIOZAKI K AND YANAGIDA M. (1991). A functional 125-kDa core polypeptide of fission yeast DNA topoisomerase II. *Mol. Cell Biol.*, **11**, 6093–6102.

TAKANO H, KOHNO K, ONO M, UCHIDA Y AND KUWANO M. (1991). Increased phosphorylation of DNA topoisomerase II in etoposide-resistant mutants of human cancer KB cells. *Cancer Res.*, **51**, 3951–3957.

VASSETZKY YS, ALGHISI G-C AND GASSER SM. (1995). DNA topoisomerase II mutations and resistance to anti-tumour drugs. *BioEssays*, **17**, 767–774.

WANG JC. (1985). DNA topoisomerase. *Annu. Rev. Biochem.*, **54**, 665–697.

WASSERMAN RA AND WANG JC. (1994*a*). Analysis of yeast DNA topoisomerase II mutants resistant to the antitumour drug amsacrine. *Cancer Res.*, **54**, 1795–1800.

WASSERMAN RA AND WANG JC. (1994*b*). Mechanistic studies of amsacrine-resistant derivatives of DNA topoisomerase II. *J. Biol. Chem.*, **269**, 20943–20951.

WASSERMAN RA, AUSTIN CA, FISHER LM AND WANG JC. (1993). Use of yeast in the study of anticancer drugs targeting DNA topoisomerases: expression of a functional recombinant human DNA topoisomerase II alpha in yeast. *Cancer Res.*, **53**, 3591–3596.

WATT PM AND HICKSON ID. (1994). Structure and function of type II DNA topoisomerases. *Biochem. J.*, **303**, 681–695.

WORLAND ST AND WANG JC. (1989). Inducible overexpression, purification, and active site mapping of DNA topoisomerase II from the yeast. *Saccharomyces cerevisiae*. *J. Biol. Chem.*, **264**, 4412–4416.